# Report of a Case of Abdominal Hysterectomy

**Published:** 1894-10

**Authors:** J. Cummings

**Affiliations:** Austin, Texas


For Texas Medical Journal.

### REPORT OF A CASE OF ABDOMINAL HYSTERECTOMY.

BY J. CUMMINGS, M. D., AUSTIN, TEXAS.

[Read before the Travis County Medical Society.]

MRS. B., age 36 years, was visited first by me September 16, 1893. She was confined to her bed with slight fever. Some sanguineous flow existed coming from the womb. She had been in this condition several weeks before I saw her. Vaginal examinations showed enlargement of the uterus, which was also tender to the touch. I suspected pregnancy with threatened abortion, and at first tried to allay irritability and check flow, and relieve the fever. After several days treatment on this line, the dischages being more or less offensive, and fever no better, I then began to use means to empty the womb. The anterior enlargement of the womb made dilatation and curette of no avail, it being impossible to reach the suspected mass within. Packing the interior of the womb with strips of gauze saturated with carbolized water was then resorted to.

At the second or third packing, not less than nine feet of a strip of gauze near one inch wide was finally placed in the cavity of the womb. This was followed by the expulsion of a very hard afterbirth, with very offensive odor, which had evidently been in the uterus for some time. Aided by some intra-uterine aseptic treatment, she was soon able to get up and did her house work for a time. However, during February, 1894, she again had fever and sent for me. The bloody and offensive discharge had returned. I again inserted carbolized gauze into the womb with some benefit, but she remained in her bed several weeks. Digital examination showed the growth in the anterior wall to be larger, as well as the entire womb. Great soreness on pressure existed. I now despaired of curing my patient without an operation probably involving the extirpation of the womb and its appendages, and so informed her and her husband. Dr. W. J. Mathews, at my request, visited the lady with me, and after careful digital examination concurred with me in recommending an operation. Three months elapsed before I was notified by her husband that they were ready to have the operation performed. She had continued to lose strength, and began to suffer more than usual. She was in bed looking haggard and pale, her countenance indicated her previous suffering. Physical examination showed no change for the better. Every detail was arranged for the operation, and Monday, May 21st following, assisted by Drs. Mathews, Graves, Wooten and McLaughlin an exploratory incision was made, and on inserting the first two fingers of the right hand into the abdominal cavity, the hard upper margin of the fibroid was first felt. The condition was unmistakable. While the anterior wall was the seat of the growth, yet the whole uterus, except the neck was enlarged and changed in structure. Both ovaries were enlarged and showed evidence of the result of inflammatory action. The tube on one side was shortened and the ovary laid against the body of the womb, bending the tube downward and forward. The lateral structures were ligated in successive sections and cut off leaving all ligatures long. The return flow next to the womb was checked by applying forceps. The neck was ligated by a double silk ligature tied on opposite sides, left long as all ligatures were; bleeding was slight. The abdomen after taking out the womb and appendages was flushed with warm water that had been previously boiled. All ligatures were passed through Douglas' cul-de-sac, through into the vagina (which had been previously rendered as aseptic as possible) for the purpose of drainage. No other means of carrying off discharges from the site of the operation was used. The stump or neck of the womb was dropped back into the pelvis, and the abdominal wound stitched by deep seated silk interrupted sutures, as in ordinary abdominal operations. There was constant drainage along the ligatures. As late as the 11th day some accumulation of secretions from the inside occurred, due to some temporary obstruction, causing stoppage of the discharge. All symptoms subsided as soon as the flow was re-established, which came spontaneously. Scarcely any fever followed. During a few evenings there was some rise of temperature, always subsiding during the night. The temperature in the morning being never higher than 101 degrees. As is usual with me in such cases, I gave every six hours enemas of either warm milk, with a little salt and water added, or malted milk; limiting the nourishment and water given by the mouth to very small quantities, preserving the stomach which is invariably irritable after these abdominal operations. In this case on the fourth day I felt sure she would recover. Her condition warranted such an opinion, and subsequent history has shown the same to have been correct. She is at the present time up and assisting in her house work.

"Schmall examined the condition of the endometrium in fifteen cases in which hysterectomy had been performed for fibroids. His conclusions are, that in cases of fibromata which protrude into the uterine cavity, the mucous membrane covering the tumor atrophies, while that portion opposite the tumor becomes hypertrophied." "The hypertrophied condition, he finds due to a morbid growth of epithelial structure. This explains the liability of such alteration in the mucous membrane to take on a malignant growth."

Popoff has examined many ovaries and tubes removed with fibromata, and says: "In fibromata of the uterus, the ovaries are almost always the subject of more or less extensive changes, including the tunica albuginea, the interstitial tissue, and the follicles." There is proliferation of the interstitial tissue, leading to increase of the volume of the ovary.

There has been an old idea that fibroid tumors cease to grow after the menopause. One author (Johnson) says: "The rule has more exceptions than is generally supposed, and that when they continue to grow after the menopause they persue a more disastrous course than before. They more frequently become cystic, calcareous, or have abscess develop in them."

It is said that if the above considerations prove true, it must follow that they furnish additional indications for more frequent and earlier resort to the radical operation. Martin speaks of the perfection of vaginal drainage, after abdominal hysterectomy, in which he uses this mode of drainage, and speaks of its advantage over ventral fixation. The mortality by this method has been greatly decreased, and recoveries are quicker and more perfect.

Sutton, of Pennsylvania, reports in the *New York Medical Journal*, May 5th, seventeen extirpations of the womb, with but one death, about six per cent. mortality. He refers to having left the ligatures used in ligating the uterine arteries long, and passing them into the vagina, with good results. It seems at least one high authority is using, or has used, the method I originally instituted, without knowledge of its use by another. However, he did not leave all the ligatures required in any given operation long, and seemed to have not used them so completely and avowedly for drainage purposes as I had done. So far as I know, he has used this method only in extirpation of the womb, while I, as you all know, began its use in operations on the ovaries and tubes, extending it later to abdominal hysterectomy.

The use of the vagina for drainage after pelvic operations, has been seriously questioned by many, fearing septicæmia, as they claim a channel is left whereby septic germs may enter the peritoneum from this source. Prof. Paine, of Galveston, in his criticism of my paper read last April before the Texas State Medical Association, where I reported several cases wherein I had used this method of vaginal drainage, having left all ligatures long, and passed them through Douglas' cul-de-sac into the vagina, referred to J. Marion Sims' effort to use vaginal drainage in his day, and that all effort at vaginal drainage had proved a failure and had been abandoned by the profession. This was before the days of aseptic surgery, and I am surprised that in the face of the progress of vaginal drainage in abdominal and vaginal hysterectomy, a gentleman of such high attainments should so express himself. Abdominal or ventral fixation of the stump after hysterectomy has almost been abandoned by the profession, and the vagina is almost exclusively and most efficiently used for drainage in all forms of this operation, one operator, over a year ago, having operated fifty times, with only two deaths,—a four per cent. mortality. The best results have been by this method of drainage, and in my opinion will continue to be, until, before long, no other method, except some form of vaginal drainage, will be used not only in extirpation of the womb, but where the ovaries and tubes are so badly diseased as to require some method of drainage also. I have left all ligatures long in five cases, and used them through the vagina for drainage, all but the case here reported being operations on either one ovary or both, with tubes, with recovery in every case, and perfect drainage as well. Each ligature serves as a guide to every cut surface to one common and most dependent point, avoiding pockets and collections of pus, as well as ligature sinuses, except where you have the ligaature under perfect control. In no case has there been the least tendency to septicæmia, the temperature never having reached a high point, and no chill has occurred in any case. Many unsatisfactory recoveries have been reported, where the patient has been said to have been left in as bad a condition as before the operation, abdominal pains continuing. In my cases, where sufficient time has elapsed to tell definitely, I have had no such results, every case regaining their accustomed health, and have suffered no more at the site of the operation.

